# Tieing together loose ends: telomere instability in cancer and aging

**DOI:** 10.1002/1878-0261.13299

**Published:** 2022-08-16

**Authors:** Gustavo Borges, Mélanie Criqui, Lea Harrington

**Affiliations:** ^1^ Molecular Biology Programme, Institute for Research in Immunology and Cancer University of Montreal QC Canada; ^2^ Departments of Medicine and Biochemistry and Molecular Medicine University of Montreal QC Canada

**Keywords:** aging, cancer, genome instability, senescence, telomeres, telomerase reverse transcriptases

## Abstract

Telomere maintenance is essential for maintaining genome integrity in both normal and cancer cells. Without functional telomeres, chromosomes lose their protective structure and undergo fusion and breakage events that drive further genome instability, including cell arrest or death. One means by which this loss can be overcome in stem cells and cancer cells is via re‐addition of G‐rich telomeric repeats by the telomerase reverse transcriptase (TERT). During aging of somatic tissues, however, insufficient telomerase expression leads to a proliferative arrest called replicative senescence, which is triggered when telomeres reach a critically short threshold that induces a DNA damage response. Cancer cells express telomerase but do not entirely escape telomere instability as they often possess short telomeres; hence there is often selection for genetic alterations in the *TERT* promoter that result in increased telomerase expression. In this review, we discuss our current understanding of the consequences of telomere instability in cancer and aging, and outline the opportunities and challenges that lie ahead in exploiting the reliance of cells on telomere maintenance for preserving genome stability.

## Graphical abstract

Normal cells and stem cells rely on genome integrity to survive repeated cell divisions. In aging and cancer, the inability to maintain telomeres can lead to telomere erosion and a DNA damage response (DDR). Telomerase insufficiency induces phenotypes that can overlap between aged cells and cancer cells, or that can even further drive age‐associated cancer (e.g. the senescence‐associated secretory phenotype, or SASP).

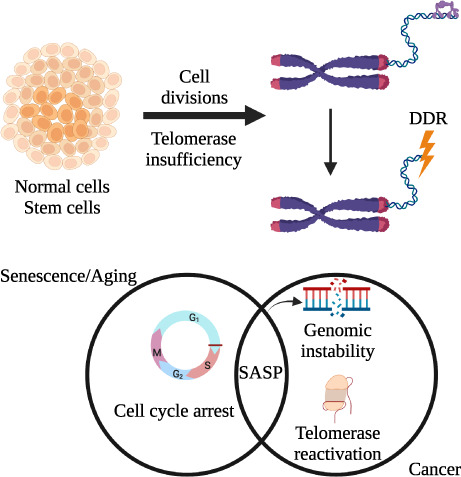

**Abbreviations**
ALT: alternative lengthening of telomeres
AML: acute myeloid leukemia
ATR: ataxia telangiectasia and Rad3‐related kinase
ATRA: all‐trans retinoic acid
AZT: azidothymidine (antiretroviral drug used to treat HIV)
BRAF: serine/threonine‐protein kinase B‐raf
BRCA1: breast cancer type 1 susceptibility protein
BRCA2: breast cancer type 2 susceptibility protein
c16orf72: chromosome 16 open reading frame 72
CpG island: regions in the genome that contains repeated CG dinucleotides
CTC1: CST complex subunit CTC1
DDR: DNA damage response
Dnmt3b: DNA methyltransferase 3B
EGFR: epidermal growth factor receptor
ETS: erythroid transformation specific
EV: extracellular vesicles
FDA: Food and Drug Administration
H3K27: histone H3, lysine 27
hTR: human telomerase RNA component
kbp: kilobase pairs
mESC: murine embryonic stem cells
miRNA: micro RNAs
nt: nucleotides
NSCLC: non small cell lung cancer
p21: cyclin‐dependent kinase inhibitor 1 or CDK‐interacting *protein* 1
p53: cellular tumor antigen p53
POT1: protection of telomeres protein 1
PRC2: polycomb repressive complex 2
RAP1: telomeric repeat‐binding factor 2‐interacting protein 1 or repressor activator protein 1
Rb: retinoblastoma‐associated protein
REV7: mitotic spindle assembly checkpoint protein MAD2B
SASP: senescence‐associated secretory phenotype
SHLD1: shieldin complex subunit 1
SHLD2: shieldin complex subunit 2
SHLD3: shieldin complex subunit 3
STN1: CST complex subunit STN1
TAPR1: telomere attrition and P53 response 1 protein (alias for c16orf72)
TEN1: CST complex subunit TEN1
TERT: telomerase reverse transcriptase
THOR: TERT Hypermethylated Oncological Region
TIMP: tissue inhibitors of matrix metalloproteinases
TIN2: TRF1 interacting nuclear factor 2 protein
TPP1: telomere protection protein 1, encoded by adrenocortical dysplasia homolog ACD
TRF1: telomeric repeat binding factor 1 protein
TRF2: telomeric repeat binding factor 2 protein
VEGF: vascular endothelial growth factor
WT: wildtype

## 1 Introduction

Telomeres are nucleoprotein complexes at the ends of chromosomes that protect cells from the loss of genetic information and from chromosomal fusions and other abnormalities caused by the untimely activation of the DNA damage response. In most eukaroytes, telomeric DNA is comprised of long tracts of a conserved, G‐rich repeat that is represented by the general sequence T_x(_A_x)_G_x_(C). In humans and mice, telomeres consist of 5′‐TTAGGG‐3′ repeats that vary in length between < 20 kbp in humans and can exceed 50 kbp in mice [1, 2]. The conserved nature of telomeres suggests that they play an essential role that originated in our primitive ancestors [3, 4, 5, 6].

Telomeric DNA repeats are double‐stranded but end with a single‐stranded, G‐rich 3′ overhang, typically 150 nucleotides (nt) in length. As this overhang contains the same sequence as telomeric DNA that is more distal to the chromosome terminus, it can loop back and invade the double‐stranded DNA region to form a displacement loop (D‐loop); when localized at the telomere, this structure is also called a telomere loop (T‐loop) [7, 8, 9, 10]. This unusual looped DNA structure serves as a docking site for shelterin, a six‐subunit complex (containing TRF1, TRF2, TIN2, RAP1, POT1 and TPP1) that binds to telomeres [11, 12]. Shelterin interacts together with other complexes, such as CST (CTC1, TEN1, STN1) [13, 14, 15, 16] and the multi‐subunit complex, shieldin (SHLD1, SHLD2, SHLD3, REV7) [17] to regulate telomere DNA replication and suppress inappropriate telomere processing or recombination [9, 18, 19, 20, 21]. In addition to structural protection there is a need for telomere replenishment because the 5′ terminus of each DNA molecule is incompletely replicated. This so‐called ‘end replication problem’, together with nucleolytic trimming of chromosome ends, leads to progressive loss of telomeric sequence during each round of DNA replication [22, 23, 24, 25].

Without a means to counterbalance this loss, inexorable telomere erosion results in a critically short threshold of telomere length that triggers a DNA damage response and cell cycle arrest (in normal cells that retain an intact DNA damage checkpoint) or cell death due to mitotic catastrophe. In human fibroblasts in culture, this response limits cellular lifespan – a phenomenon called replicative senescence or the Hayflick limit [26, 27]. *In vivo*, adult somatic cells from highly proliferative or minimally proliferative tissues often undergo telomere attrition at a similar rate [28]. In many eukaryotic cells, including stem cells and cancer cells, this loss is often counteracted by the expression of the telomerase reverse transcriptase (*TERT*) and its integral RNA template component hTR (encoded by *TERC)*. This telomerase complex and its associated co‐factors are recruited to telomeres during S‐phase and serve to elongate the 3′ telomeric overhang [29, 30, 31]. Despite retaining low levels of telomerase activity, telomere erosion nonetheless occurs in most human stem and progenitor cells [32, 33].

Emerging evidence underscores that there are important similarities in how telomere integrity affects aging, normal stem cells (as discussed below), and cancer cells (as discussed later). In this review, our goal is to highlight these common themes and the opportunities and challenges that lie ahead in exploiting this information in the development of new cancer treatments.

## 2 Telomeres in aging and stem cells

Telomere shortening is one of the main hallmarks of aging and is commonly observed in most cell types [34]. Telomere shortening can occur through the well‐known end‐replication problem or as a result of independent stochastic events.

In aging, the impact of telomere erosion on human and organismal lifespan is an extant question. While telomere length decreases with age in all cell types, the average telomere length varies between species significantly and does not strictly correlate with lifespan [35, 36]. One such example is that of mice and humans; mice have longer telomeres than do humans but a shorter lifespan [2]. However, mice that lack sufficient levels of telomerase activity undergo telomere erosion and eventually reach an average length similar to some normal human tissues. As such, they provide a powerful genetic model in which to assess the role of eroded telomeres in aging and cancer [37, 38]. In marine animals, it is the body size of marine mammals, rather than telomere length, that dictates life expectancy [39]. In contrast, in some species, such as the wild Soay sheep, shorter average leukocyte telomeres are associated with an increased risk of early mortality [27, 40, 41]. In humans, females have been reported to possess longer blood cell telomere lengths than do males [42], and shorter average telomere lengths correlate with several human disorders [43, 44].

Other biological clocks have been established that reflect chronological age, such as the methylome clock, which is defined as age‐associated changes in the pattern and extent of methylated DNA [45, 46]. The telomere clock appears to more closely reflect a ‘replication clock’ rather than a chronological clock [46, 47, 48]. For example, only a weak correlation between telomere length and chronological age has been found in two independent studies [48, 49]. A relationship between the methylated status of 140 CpG islands and telomere length has also been described but might more closely reflect the population doubling time of the cell population [48].

### 2.1 Telomere erosion: a harbinger of senescence

**Fig. 1:**
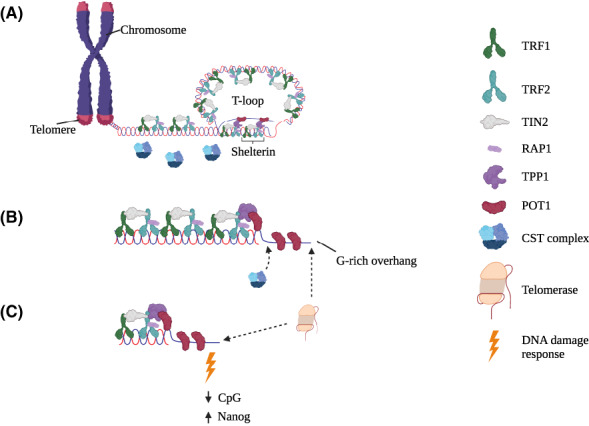
Overview of mammalian telomere structure. (A) Schematic representation of the chromosome end, or telomere in mammals. Telomeric DNA is composed of the hexanucleotide sequence TTAGGG with a single stranded G‐rich 3′ overhang that is looped back into the telomeric DNA to form a telomeric loop (T‐loop). The shelterin complex (composed of the proteins TRF1, TRF2, TIN2, RAP1, TPP1 and POT1) protects telomeres from being recognized as a DNA double‐stranded break. (B) During the S‐phase of the cell cycle, the T‐loop is opened and the G‐rich strand becomes accessible for extension by telomerase. After telomerase has extended the G‐rich strand, the CST complex (composed of the subunits CTC1, STN1, TEN1) together with the DNA replication machinery carries out fill‐in DNA synthesis of the complimentary C‐rich strand. (C) Critically short telomeres exhibit defects not only in telomere integrity (due to a DNA damage response) but can also perturb the epigenetic state (reducing CpG methylation and increasing Nanog expression). [Colour figure can be viewed at wileyonlinelibrary.com]

**Fig. 2:**
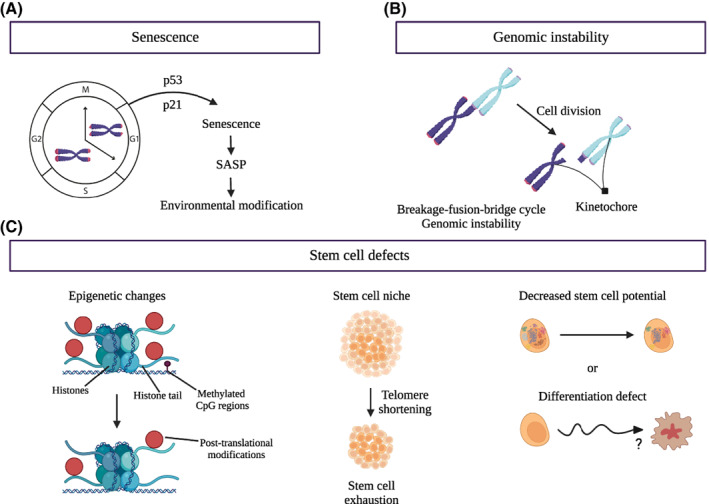
Consequences of telomere shortening. (A) Senescence is referred to as an irreversible exit from the cell cycle. This state can occur via a variety of mechanisms that include eroded telomeres. When telomeres reach a critically short threshold, cells undergo telomere‐induced (or replicative) senescence, which is triggered by p53 and its downstream effector p21, a cyclin‐dependent kinase inhibitor that arrests the cell cycle. Senescent cells can secrete factors that modify neighbouring tissues and cells and enhance aging, via a process termed the senescence‐associated secretory phenotype (SASP). (B) If a cell bypasses senescence and continues to divide, telomere fusions can occur, leading to a cycle of chromosome breakage‐fusion‐bridge formation and genetic instability. (C) Epigenetic modifications are also linked to telomere shortening. Such modifications can influence transcriptional activity and cell fate. In the left image, DNA wrapped around the histones (represented by the green spheres) may contain extensive post‐translational modifications (red sphere) on the histone tail, and the DNA itself can be methylated (small black sphere). Murine embryonic stem cells are particularly sensitive to epigenetic modifications driven by telomere shortening, which can reduce stem cell renewal potential (middle image). Telomere shortening can also affect the ability of stem cells to maintain a functional pluripotent state and/or differentiated state (right image). [Colour figure can be viewed at wileyonlinelibrary.com]

Telomere attrition to a critical threshold length that is no longer able to sustain telomere integrity is one of several known inducers of cellular senescence [50]. This type of senescence, termed telomere‐induced or replicative senescence, can be triggered by the well‐known tumour suppressor p53 and its downstream effector p21, a cyclin‐dependent kinase inhibitor that represses the cell cycle (as discussed in an accompanying article in this special series [51, 52]). Mathematical models and studies of budding yeast have suggested that the shortest telomeres are responsible for senescence induction [53, 54]. In human cancer cells, the minimum length of a functional telomere is 12 bp [55], beyond which telomere‐telomere fusions are observed. Importantly, damage at a single telomere is enough to induce cell cycle arrest in budding yeast [56] and to induce cycles of chromosome breakage‐fusion‐bridge in cancer cells [57, 58, 59, 60] (Figs 1 and 2).

Telomere shortening is not the only telomeric damage that leads to senescence. The oxidation of telomeric DNA (to form 8‐oxoguanosine) induces senescence without telomere shortening, underlining the critical contribution of the DNA damage response in this process [61]. Moreover, cellular senescence can be induced by factors other than telomere erosion, including by persistent DNA damage across the genome, oncogene activation, loss of a tumour suppressor or other stressors, and thus critically short telomeres are not the sole hallmark of senescent cells [62]. Senescence is also triggered by two different pathways, one through p53, which is reversible, and the other through Rb, which is permanent [63, 64].

The presence of senescent cells in a tissue profoundly modifies tissue function. Senescent cells secret specific small molecules or exosomes (EV) that interact with and influence the nearby environment. This phenomenon is known more generally as the Senescence‐Associated Secretory Phenotype (SASP) and is a phenotype common to many senescent states, including that induced by eroded telomeres (please see accompanying review) [51, 52, 62, 65, 66] (Fig. 2). Among the molecules secreted by senescent cells exhibiting a SASP are pro‐inflammatory cytokines that promote the recruitment of immune cells to clear the growth‐arrested cells [67]. Senescence is thought to represent a normal process for somatic cells, as the presence of the SASP stimulates tissue regeneration by encouraging stem cell proliferation and differentiation [68, 69]. This rejuvenation process is important for ensuring tissue function, as it enables growth‐arrested somatic cells to be cleared away and replaced by newly differentiated cells.

However, the accumulation of senescent cells that arise via a telomere induced DDR or other stresses can also negatively affect nearby cells, promoting aging and tissue dysfunction. For instance, the Von Zglinicki lab has shown that the xenotransplantation of senescent cells into mouse muscle or skin induces senescence in cells near the transplantation site [70]. In addition, more recent studies have shown that the accumulation of senescent cells during aging can impact cells far from their location. EV and other microvesicles in circulation in the body carry small molecules, such as miRNA, that can affect the behavior of cells along their journey. With age, the composition of such vesicles changes. For example, microRNA‐183‐5p has been shown by Davis et al. [71] to increase in microvesicles with age, resulting in the suppression of bone marrow stromal stem cell proliferation and accompanied by senescence. These and other studies suggest that the *in vivo* removal or manipulation of senescent cells could have physiologically relevant benefits in ameliorating aging and age‐associated disease [72].

As senescence is generally driven by cellular dysfunction, a logical, proposed role for senescence is to suppress cancer [73, 74]. Oncogene‐induced senescence is one such example of a suppressive mechanism (reviewed in [75]). While senescence and SASP induction is tumor‐suppressive in many contexts, it can also drive tumorigenesis (reviewed in [76]). For instance, cancer incidence increases with age, concomitant with a rise in tissue senescence [64]. Among the SASP secreted molecules, VEGF and tissue inhibitors of metalloproteinases (TIMP) promote cellular migration, including the potential of cancer cells to undergo metastasis [77, 78]. Other recent studies have shown that the presence of senescent cells, whether via perturbations in the DNA damage response or loss of telomere integrity, can modify tissue homeostasis during aging, which could also act to promote cancer [79, 80].

### 2.2 Telomeres and stem cells

Adult stem cells remain in the body throughout life, mainly in a protected environment called the stem cell niche, and can regenerate tissues via self‐renewal and differentiation. Thus, the sustained presence of functional stem cells is essential for tissue homeostasis, and the loss of the stem cell pool can lead to disease (Fig. 2).

Despite the ongoing expression of telomerase in certain stem cell and progenitor populations, telomere lengths, on average, are shorter in cells from older individuals than in cells from younger individuals. This telomere erosion can have varying effects on stem cells, from limiting their proliferation potential to inducing severe cell dysfunction [81, 82, 83]. Perturbations in stem cell function can impact the stem cell pool that is available to rejuvenate tissue, which in turn can contribute to age‐associated issues like stem cell depletion [84]. Similar age‐associated impacts on stem cells pools have been observed in diseases driven by telomere or telomerase insufficiencies. For example, some symptoms observed in dyskeratosis congenita or pulmonary fibrosis occur due to defects in the stem cell compartment and via the loss of cellular self‐renewal potential [85, 86, 87].

In some instances, these barriers to stem cell proliferation that develop with age can lead to the senescence of adult stem cells. For example, stem cell senescence has been described in bone marrow stromal stem cells and in mesenchymal stem cells [88]. Moreover, senescence impairs pluripotent cell reprogramming [89]. This limit on stem cell proliferation with age could have implications for stem cell therapy and its use of amplified stem cells. In addition, the presence of senescent cells in the stem cell niche could induce senescence in neighboring cells or could increase inflammation in the niche [71, 90, 91, 92].

In addition to limiting their capacity to proliferate, telomere attrition in stem cells can lead to the generation of dysfunctional differentiated cells (Fig. 2). For example, in aged hematopoietic stem cells, some progenitor cells differentiate inappropriately, therefore reducing the number of functional blood cells [93]. Other studies also suggest a differentiation bias during aging, i.e. the skewing of certain subpopulations in a manner that perturbs overall tissue function and resiliency [94].

Several unanswered questions remain as to the precise mechanisms by which telomeres influence stem cell behaviour. As mentioned earlier, the arrest of cell proliferation once a critical level of telomere attrition is reached is mainly instigated by DNA damage signaling. However, the signals that regulate differentiation are more complex and are tighly regulated by epigenetic mechanisms. For example, the chromatin of stem cells is known to be open and dynamic. However, during the process of differentiation, chromatin acquires repressive marks and a closed conformation, with the exception of particular lineage‐specific genetic regulatory elements, which become decorated with chromatin marks associated with either transcriptional activity or repression [95, 96]. Telomere erosion itself is also linked with marked alterations in the epigenetic state [94, 97, 98, 99]. For example, when telomere protection or telomere erosion are perturbed in mice, genome‐wide transcriptional and epigenetic alterations ensue [82, 100, 101, 102, 103, 104, 105, 106, 107, 108]. Furthermore, during senescence, or upon the distruption of telomere maintenance or Trf1 function, associated changes in the chromatin landscape occur that are linked to the dysregulation of the chromatin modifying complex, Polycomb Repressive Complex 2 (PRC2) and/or to changes in histone or DNA methylation status [81, 82, 109].

To elaborate on one such example, in mESCs with critically eroded telomeres (via prolonged propagation in the absence of *Tert*), cells fail to fully consolidate a differentiated state upon treatment with a differentiation‐inducing agent (*all‐trans* retinoic acid, ATRA). These cells are also prone to re‐acquire a transcriptional and chromatin landscape similar to that of undifferentiated cells [81, 82]. In this experimental context, the overexpression of the DNA methyltransferase Dnmt3b or the chemical/genetic modulation of PRC2 activity partially rescues the ability of *Tert* ^−/−^ mESCs to differentiate in response to the appropriate cues such as ATRA [81, 82]. Telomere erosion or perturbed telomere protection complexes are also associated with genome‐wide epigenetic alterations in budding yeast [110], which underscores the evolutionary conservation of this complex interrelationship.

A better understanding of the ways in which telomere erosion is linked to the genetic and epigenetic alterations that underlie the aging process has important implications for cancer, which leads us to the topics we discuss in the next section. For example, Ideker et al. [45] have shown that the methylome of older individuals shares similarities with the methylomes of patient‐derived cancers. Cancer stem cells share important characteristics with normal stem cells, such as unlimited proliferation, self renewal and differentiation capacity. Emerging evidence suggests that some cancer types, particularly glioblastoma and acute myeloid leukemia (AML), have reverted to a more primitive cell state via genetic and epigenetic alterations that exhibit striking similarities [111]. Thus, as seen in normal stem cells, the epigenetic alterations of cancer stem cells should not necessarily be considered in isolation from other cancer‐associated characteristics, such as short telomeres [112, 113].

## 3 Telomeres and telomerase in cancer

As mentioned above, the co‐existence of eroded telomeres and telomerase activity in cancer cells is similar to that observed during aging of stem cells. While telomerase is re‐activated in 85% of cancers [114], telomeres are relatively short in these cells [33, 115]. The upregulation of telomerase is a common occurrence in human cancers and occurs via multiple mechanisms [116]. In 10–15% of human cancers, telomerase is not reactivated, and cancer cells can survive without telomerase, usually via mechanisms that involve alternative lengthening of telomeres (ALT) (reviewed in [117]). These alternative mechanisms are more prevalent in certain types of tumours, such as in osteosarcomas [118], soft tissue sarcomas [119], glioblastoma [120] and neuroblastoma [121]. One of the most common mechanisms of telomerase upregulation in tumours is the presence of mutations at the *TERT* promoter. Indeed, mutations at the *TERT* promoter region are the most frequent, non‐coding mutation in cancer [122]. These mutations create binding motifs for the Erythroid Transformation Specific (ETS) transcription factors, which result in increased expression of *TERT* [123, 124, 125, 126]. Even a modest increase in *TERT* expression, which results in slightly more telomerase activity, is sufficient to confer a proliferative advantage. These *TERT* promoter mutations are thought to act as driver mutations because they confer a fitness advantage and thus may promote or drive cancer progression. Such mutations were first identified in two unrelated cohorts of melanoma patients [123, 124] and have subsequently been identified in subsets of glioblastoma, gliomas, thyroid cancer, hepatocellular carcinomas, bladder cancers and mantle cell lymphomas [127, 128, 129, 130].

### 3.1 TERT expression and telomere length: clinical indicators

Rare, somatic mutations within the *TERT* promoter region are also found in non‐malignant diseases, such as in idiopathic pulmonary fibrosis and aplastic anemia [131, 132]. In these instances, these mutations are often found in *cis* to the wild‐type *TERT* allele, and it is thought that that compensatory upregulation of the wild‐type allele may promote somatic gene rescue [131]. As in cancer, these mutations at the *TERT* promoter increase *TERT* transcription only marginally (by twofold or less), but nonetheless this increase is still sufficient to delay replicative senescence in human fibroblasts [123, 124, 125].

Epigenetic modifications can also increase *TERT* expression. For example, a hypermethylated region of the *TERT* promoter known as THOR (TERT Hypermethylated Oncological Region) was first detected in a group of pediatric brain tumours and later shown to drive the expression of the *TERT* locus in various tumour types, such as in brain tumours, and in bladder, breast, colon, lung and prostate cancers [133, 134, 135]. Although present in a wide range of tumour types, this hypermethylation pattern is rare in normal tissues [135], and is considered a potential tumour biomarker. As yet, the correlation between THOR hypermethylation and prognosis remains to be elucidated. In patients with bladder cancer and melanoma, THOR hypermethylation combined with *TERT* promoter mutations are associated with an increased risk of tumour progression and with a decreased progression‐free survival [136, 137]. However, THOR hypermethylation does not correlate with disease progression and/or survival in medulloblastoma, esophageal cancer or in meningioma, among other tumour types (reviewed in [138]).

Another distinguishing feature of adult cancers is their relatively short average telomere lengths, which have been linked to disease outcome. For example, patients with a range of cancers (including chronic lymphocytic leukemia, breast cancer, non–small cell lung cancer, myelodysplastic syndromes and multiple myeloma), whose leukocytes have short telomeres, have an unfavourable prognosis relative to those whose telomere lengths are within the normal range (reviewed in [139]). One might thus assume that short telomeres would always be detrimental. However in the case of esophageal cancer [140], prostate cancer [141], clear renal cell carcinoma [142], melanoma [143] and hepatocellular carcinoma [144], longer telomeres are associated with a poorer prognosis, possibly related to difficulties in replicating long telomeric tracts. As Rivera and colleagues showed, long telomeres might also be detrimental to cell fitness [145]. Using human embryonic stem cells and human induced pluripotent stem cells, the group showed that long telomeres led to the formation of C‐circles (the C‐rich single‐stranded telomeric DNA complimentary to the G‐rich strand; also a common biomarker of the ALT pathway [146]). Excessively long telomeres can lead to increased sensitivity to replication stress [145], increased sensitivity to DNA damage [147] and telomere fragility [145, 148]. Altogether, these studies suggest that there exists an optimal telomere range for cancer cell fitness, that was referred to as the telomere ‘Goldilocks Zone’ [149].

### 3.2 Therapeutic approaches to telomerase inhibition

The effects of telomerase inhibition have been widely studied in different cell types. Telomerase inhibition leads to telomere shortening, followed by a reduction in cancer cell growth and/or apoptosis [150, 151, 152]. Even in proliferating tumour cells, genome rearrangements such as chromothripsis (numerous clustered chromosomal rearrangements that arise in a single event) and hypermutational regions (kataegis) appear to be driven, in part, by telomere instability [153, 154, 155, 156].

Unfortunately, an FDA‐approved telomerase inhibitor is not yet available. Currently, numerous approaches are being pursued that target telomerase or telomere integrity in preclinical and clinical settings, including telomerase inhibitors, the targetting of telomere replication, vaccines, and other immunological approaches (reviewed in ref. [157]). The only direct telomerase inhibitor used in clinical trials so far, Imetelstat, is a modified 13‐mer oligonucleotide that shares complementarity to the human TR (telomerase RNA component) template region [158, 159]. Imetelstat competes with the telomeric DNA substrate, leading to telomerase inhibition and consequently to telomere shortening [159]. Currently, Imetelstat is being used in phase II/III clinical trials in myelodysplastic syndromes and refractory myelofibrosis. In a phase II study in patients with intermediate/high‐risk myelofibrosis, treatment with Imetelstat exhibited a better response in patients bearing the JAK2‐V617F mutation [160]. However, the molecular mechanism behind this response is unclear, and there was no significant reduction in telomere length in the patients who exhibited a partial or complete response [160].

**Fig. 3:**
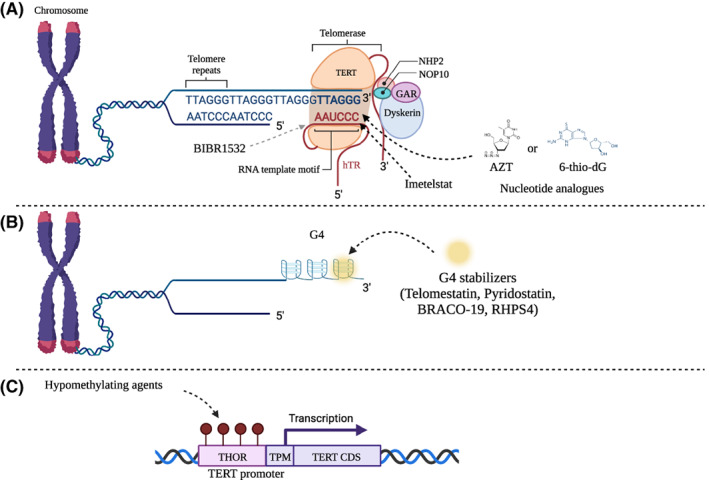
Current and emerging telomere‐targeting treatments. (A) Therapeutic strategies that target telomerase use compounds (such as Imetelstat and BIBR1532) that can directly inhibit the catalytic core of the telomerase enzyme, or they may also affect telomere DNA replication (such as the nucleotide analogue AZT or the nucleoside analogue 6‐thio‐dG). The telomeric DNA sequence is indicated (TTAGGG repeats in the 5′ to 3′ direction). Telomerase is a multi‐subunit complex that contains TERT (orange), the telomerase RNA (hTR), and associated subunits (GAR, NOP10, NHP2, and dyskerin). For simplicity only 1 telomeric repeat of the hTR template motif is shown; the full template contains 1.5 telomeric repeats (CAAUCCCAAUC) that is reverse‐transcribed in an iterative process. (B) More recently, compounds that affect telomere structure, such as G‐quadraplex (G4) stabilizers, have been investigated, as they induce immediate cell death upon telomere uncapping and do not exhibit a therapeutic lag, as is the case with telomere attrition. The telomeric G‐quadruplexes are located at the 3′ end of the G‐rich strand. The yellow circle represents the G4‐stabilizers as indicated. (C) As the expression of *TERT* is regulated by DNA methylation status (through the THOR region), chromatin‐modifying agents might also have therapeutic benefit in the treatment of telomerase‐positive tumours. THOR stands for TERT Hypermethylated Oncological Region. Red spheres indicate methylated CpG dinucleotides at the THOR region that may be a potential target site for the use of hypomethylating agents. TPM indicates the promoter region where the *TERT* promoter mutations are commonly found, upstream of the coding sequence (TERT CDS). [Colour figure can be viewed at wileyonlinelibrary.com]

Another inhibitor that is not clinically approved but is widely used in preclinical models is the small molecule BIBR1532, a synthetic and non‐competitive inhibitor that shares some similarities to non‐nucleosidic inhibitors of HIV1 reverse transcriptase [161, 162]. The mechanism‐of‐action of this small molecule occurs via the non‐competitive inhibition of the catalytic site [162]. BIBR1532 binds to the C‐terminal region of TERT, in a hydrophobic motif named FVYL, that is close to the region important for the interaction of TERT with telomerase RNA (TR) [163]. BIBR1532 showed promising effects in preclinical models of solid and hematological malignancies [164, 165, 166, 167, 168]. Other telomerase inhibitors have also been found to induce telomere‐induced cell death in cell‐based models, including the nucleoside analogues azidothymidine (AZT) [169, 170] and 6‐thio‐dG [171]. Several compounds that specifically destabilize telomeric DNA have also been identified that induce apoptosis in human cancer lines, such as pyridostatin, telomestatin, BRACO‐19 and RHPS4 [172, 173, 174, 175] (Fig. 3). Also, peptide vaccines against TERT are under active investigation. One of these vaccines, called GV1001, is already in phase III clinical trials (see refs [157, 176] for a more detailed information on immunotherapies against telomerase).

### 3.3 Therapeutic challenges to modulation of telomerase activity

As telomere erosion is one of the hallmarks of aging and is associated with other deleterious age‐associated phenotypes [177], one strategy being considered is telomerase reactivation. The re‐expression of telomerase reverse transcriptase in normal human cells is sufficient to avert senescence and leads to the capacity to divide indefinitely, called immortalization [178, 179]. Immortalized cells can maintain a relatively stable karyotype even after prolonged culture [180, 181, 182]. However, reactivation of telomerase can be detrimental in other contexts. For example, in *Tert* null mice bred for successive generations until telomeres reached a critically eroded state, *Tert* reintroduction led to a marked increase in tumorigenesis [183]. Thus, while the reactivation of telomerase rescues phenotypes associated with aging in certain cell‐based and animal models [87, 184, 185, 186], it could have deleterious consequences if used as a therapy in older individuals who have acquired somatic mutations in blood or other tissues or in those with genetic predispositions that affect genome integrity (e.g. familial cancer syndromes).

Conversely, what are the prospects for telomerase inhibition as a viable treatment for cancer? The crystal structure of TERT shows that the enzyme has a catalytic core that is similar to HIV and should, in principle, be druggable [187, 188, 189, 190, 191]. Aside from the usual issues of bioavailability and toxicity, there are at least two challenges that arise with the clinical use of a telomerase inhibitor. The first challenge is the possibility of drug resistance. So far, no study has established whether genetic alterations in telomerase would lead to resistance to Imetelstat (currently in phase II/III clinical trials) or BIBR1532 (used in preclinical studies) [168, 192, 193, 194]. It is possible that amino acid alterations in TERT or nucleotide substitutions in hTR could promote drug resistance; however, many of these substitutions would also be likely to reduce enzyme activity [195, 196]. For example, in TERT, mutations at the FVYL pocket (the predicted binding site of BIBR1532) reduce telomerase activity [163]. In terms of disease, there are known alterations in *TERT* that are generally loss‐of‐function mutations and that are found in patients with telomere biology disorders, such as dyskeratosis congenita and pulmonary fibrosis [197, 198, 199]. It is worth mentioning that BIBR1532 is an allosteric inhibitor [163] and, thus, we cannot rule out the possibility that mutations outside the FVYL pocket might affect sensitivity to BIBR1532 inhibition. If resistant mutations are identified, presumably the inhibitors could be refined to act against these new variants, in the same manner as there are now later‐generation EGFR or BRAF inhibitors that specifically target their respective protein variants that led to the acquired resistant phenotype [200, 201, 202].

The second challenge is the time delay required for a telomerase inhibitor to elicit cell death or arrest, that is, the fact that several cell divisions are needed before telomere erosion triggers a DNA damage response. This is known as the lag period, and evidence suggests that initial telomere length can affect the time required to elicit cell death in response to telomerase inhibition [150, 151, 152]. In other words, tumours with short telomeres would require fewer cell doublings to trigger growth arrest or cell death. By contrast, tumours possessing longer telomeres would require a prolonged treatment period before the telomeres reach a critical threshold. In one study, the removal of telomerase from engineered human tumor cells with long telomeres did not significantly impact cell proliferation or tumour formation for several months in culture, until telomeres eventually reached critically shortened lengths [203]. This study underscores the issues surrounding the lag phase, and further raises a concern that longer periods of treatment with an inhibitor might facilitate the development of drug resistance.

The third challenge in the use of telomerase inhibitors is the checkpoint status of the cancer cells. For example, cells with critically short telomeres activate a DNA damage response and, potentially, trigger cell death (e.g. via activation of the Rb or p53 checkpoint pathway) [204, 205]. Conversely, if a tumor lacks the p53 checkpoint, the cells can continue to proliferate at the expense of genomic instability [205]. Hence, the use of telomerase inhibitors in tumors p53‐deficient could, in principle, increase the mutational burden of the tumors.

One approach to address these issues, which is now commonly employed in cancer treatment, is to combine telomerase inhibitors with inhibitors that target a gene or network whose function is critical for proliferation in the absence of telomerase. This approach of targetting synthetic‐sick‐lethal (SSL) interactions might shorten the lag period required to elicit tumour cell death upon telomere erosion and forestall the emergence of drug resistance. This approach has been used successfully in the development of poly‐ADP‐ribose polymerase (PARP) inhibitors, which are specifically lethal to tumour cells with BRCA1/2 mutations [206, 207]. Indeed, the factors to which cells with eroded telomeres are sensitized overlap with many of the other hallmarks of aging. One example of a newly identified target that is SSL with telomerase loss is the previously unannotated gene C16orf72/TAPR1 [208]. C16orf72/TAPR1 acts to taper p53 activation in response to eroded telomeres, and its loss was also identified as SSL in the presence of oxidative damage or DNA damage, and in a genome‐wide screen for deletions that sensitize cells to ATR inhibition [209, 210, 211].

Telomerase inhibitors might also be SSL with epigenetic regulators. As an example, murine stem cells with eroded telomeres are unable to stably differentiate. Inhibitors of the enzyme that deposits methyl groups on H3K27, PRC2, further exacerbate this unstable differentiation state, and inhibitors that repress the de‐methylation of H3K27me3 partially rescue the unstable differentiation phenotype [82]. Thus, one might imagine that treatments that target the epigenetic vulnerabilities of cancer, many of which are now in clinical trial, could be effective in combination with telomerase inhibitors. The only exception would be tumours that do not rely on telomerase but instead use ALT, in which case telomere replication fidelity or stability could be targeted rather than telomerase itself.

## 4 Conclusions and perspectives

Evolutionary studies in multiple organisms, including mammals, have established that telomerase and telomere‐associated factors are under positive selective pressure [212, 213, 214, 215, 216, 217]. Scientists examining this question posit that such selective pressures could arise as a result of adaptive mechanisms [217, 218]. This is also a relevant question with respect to age‐associated diseases, including cancer, whose incidence increases later in life after the reproductive period. Thus, scientists have pondered whether short telomeres later in life are the result of antagonistic pleitropy (i.e. a trait under selective pressure to be beneficial earlier in life, that later in life that becomes deleterious) [219], or of other adaptive measures independent of early‐life selection. Interesting models have been proposed to account for how mammals have evolved to cope with counterselective pressures (e.g. long versus short telomeres) in relation to aging and cancer [73]. In this post‐genomics era, there will now be an opportunity to put some of these theories to test at the molecular level, and to determine how the modulation of these selective pressures during cell/tissue aging and cancer can be exploited therapeutically to ameliorate disease.

We are at a watershed in our understanding of the molecular underpinnings of aging and cancer. The ongoing challenge is to understand how to best apply these new insights in the diagnosis, treatment, and management of cancer and other age‐associated diseases. Indeed, age is the largest single risk factor for cancer (cruk.org/cancerstats). Therefore, interventions that ameliorate age‐associated disease must be considered carefully in the context of cancer incidence and prognosis. These questions are at the nexus of the challenges and opportunities that lied ahead for those pursuing the mechanisms of telomere integrity, aging and cancer.

## Conflict of interest

The authors declare no conflict of interest.

## Author contributions

GB, MC and LH wrote and edited the manuscript. GB, with input from MC, generated the figures, which were edited by LH.
